# Nanogenerator Neuromodulation to Enable Locomotion Rehabilitation for Spinal Cord Injury via Epidural Electrical Stimulation

**DOI:** 10.1002/advs.202501425

**Published:** 2025-05-23

**Authors:** Cong Li, Yizhu Shan, Shihao Zheng, Puchuan Tan, Yuan Bai, Engui Wang, Lingling Xu, Ruizeng Luo, Shengyu Chao, Jing Huang, Pengyu Ren, Zhou Li, Hongqing Feng

**Affiliations:** ^1^ Department of Neurosurgery Second Affiliated Hospital of Xi'an Jiao Tong University Xi'an Shaanxi 710004 China; ^2^ College of Materials Science and Opto‐Electronic Technology University of Chinese Academy of Sciences Beijing 101400 China; ^3^ Beijing Institute of Nanoenergy and Nanosystems Chinese Academy of Sciences Beijing 101400 China; ^4^ Institute of Chinese Materia Medica, China Academy of Chinese Medical Sciences Beijing 100010 China; ^5^ Institute of Forensic Science of Tianjin Public Security Tianjin 300384 China; ^6^ Institute of Medical Artificial Intelligence Second Affiliated Hospital of Xi'an Jiaotong University Xi'an Shaanxi 710061 China

**Keywords:** epidural electrical stimulation, nanogenerator, neuromodulation, rehabilitation, spinal cord injury

## Abstract

Spinal cord injury (SCI) is a severe neurological disease, often accompanied by impaired lower limb motor function and muscle atrophy. Epidural electrical stimulation (EES) has been demonstrated promising for SCI therapy in ways of rehabilitation by facilitating the recovery of lower limb motor abilities. However, EES necessitates a considerable consumption of electrical energy and exhibits large individual differences in treatment. Nanogenerators (NGs) based on a novel power generation technology, are capable of transforming mechanical energy into electrical power. This mechanic‐driven electrical stimulation has been reported effective in several types of neuromodulations, but not in EES to enable SCI rehabilitation. This study explores the efficacy of a hybrid‐NG (H‐NG) to elicit hindlimb locomotion in rats via EES on the spinal cord, in comparison with a commercial stimulus generator (SG). The results reveal that H‐NG can activate the spinal cord and induce hindlimb locomotion with much lower electrical parameters and much smaller individual differences than SG. In addition, benefiting from the miniature size of the H‐NG, an implantable EES system is constructed in vivo, enabling a self‐driven and rational‐controlled EES pattern. The proposed H‐NG‐based EES system provides a new strategy for optimized and personalized treatment for SCI patients.

## Graphical abstract

A hybrid nanogenerator (H‐NG) has been developed to be applied in epidural electrical stimulation (EES). Compared with a commercial stimulus generator (SG), the H‐NG can elicit hindlimb locomotion in rats with much lower electrical parameters and much smaller individual differences. The proposed H‐NG‐based EES system provides a promising treatment technology for spinal cord injury (SCI) patients.

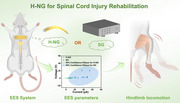

## 1 Introduction

Spinal cord injury (SCI) is a serious neurological damage, predominantly caused by traumatic factors, for instance, motor vehicular crashes, falls, or sports‐related injuries. According to the systematic review by Lu et al, the overall incidence rate of SCI globally was 23.77 per million people in the past two decades.^[^ ^1^ ^]^ People with SCI frequently suffer from lower limb motor impairment and muscular atrophy, which hinder their ability to ambulate.^[^ ^2^ ^]^ These difficulties inflict severe physical and psychological distress and drastically impair the patient's quality of life. Despite advancements in surgical procedures and pharmacological interventions,^[^ ^3^, ^4^ ^]^ the majority of patients with SCI are not able to achieve significant functional restoration. The severance of nerve fascicles, disruption of electrical signaling transmission, and the inadequate regeneration of central nervous system cells following SCI are pivotal obstacles to recovery.^[^ ^5^, ^6^ ^]^ Because electrical activity is a fundamental attribute of the central nervous system, the potential of electrical stimuli in neural repair represents a critical avenue in the field of SCI therapy.

Since the 1940s, when researchers first observed that external electric fields could prompt neuron growth and axonal extension, electrical stimulation has become an important therapeutic strategy for nerve damage. Over the past several decades, a wealth of promising outcomes have been achieved.^[^ ^7^, ^8^, ^9^ ^]^ Specifically, epidural electrical stimulation (EES) has emerged as a prevalent technique for the management of SCI. The operation process involves the surgical placement of electrodes adjacent to the spinal cord, and the stimulation of the spinal cord via the electrodes within the epidural space. In the 1950s, this technique was first employed for the management of chronic pain. In recent years, EES has been applied in patients with SCI‐induced paralysis to help them regain motor functions via rehabilitation.^[^ ^10^, ^11^, ^12^, ^13^ ^]^ Furthermore, it has been evidenced that even after the cessation of EES post‐neurorehabilitation, patients exhibit continued enhancement in their motor abilities and SCI recovery.^[^ ^14^, ^15^ ^]^

To carry out EES, necessitates a consistent energy supply. The evolution of energy technologies may introduce innovative advances to EES therapy. Nanogenerators (NGs), an emerging energy generation technology, can convert diverse ambient mechanical energies into electrical output. Since the invention of the first NG in 2006,^[^ ^16^ ^]^ various types of NGs have been developed over the past decade, leveraging principles including piezoelectric,^[^ ^17^ ^]^ triboelectric,^[^ ^18^ ^]^ and thermoelectric^[^ ^19^ ^]^ effects. NG devices are noted for their self‐powered feature, extensive material versatility, cost‐effective manufacturing, simple yet sophisticated device design, and tunable properties. To date, many electrical stimulation systems enabled by NG have been developed.^[^ ^20^, ^21^, ^22^ ^]^ Specifically, NGs have been utilized in SCI therapy, further expanding their utility in biomedical applications. For instance, Wu et al. used a triboelectric nanogenerator (TENG) for electroacupuncture, applying bidirectional continuous current to two potent acupoints in rats through needles. This therapeutic approach significantly improved the viability of ventral horn neurons and suppressed astrocyte activation at the injury site, demonstrating profound neuroprotective effects in rats with SCI.^[^ ^23^ ^]^ Besides, Lu et al. devised a biomimetic Z‐structured TENG (BZ‐TENG) that converts the kinetic energy from joint actions into electrical energy following implantation. The BZ‐TENG delivered electrical stimulation to the injury site and was demonstrated effective in the promotion of SCI healing in various aspects.^[^ ^24^ ^]^ Despite these inspiring studies, the efficacy of NG application in EES has not yet been investigated. Since EES requires a higher output parameter, it raises a challenge for NG applications in EES, especially in vivo. The development of an implantable self‐powered EES system and the comprehensive therapeutic impact of NG in SCI therapy are areas that require further exploration.

Apart from the dependence on electrical energy devices, EES has another shortcoming impeding its wider application, that is, individual differences.^[^ ^5^, ^10^, ^25^ ^]^ Both animal and clinical studies have revealed large variability in motor and physiological responses to stimulus parameters. In rats, it was discovered that effective stimulation parameters varied from treatment sites, electrode configurations, voltages, currents, frequencies, and a multitude of other elements.^[^ ^23^, ^26^ ^]^ In patients, Wagner et al. helped three SCI patients regain motor function in 2018. To get equivalent vastus lateralis responses, EES frequencies of 120, 90, and 40 Hz were used, with corresponding currents of 2.8, 13, and 9.3 mA to activate the left‐leg tibialis anterior.^[^ ^10^ ^]^ Gill et al.^[^ ^27^ ^]^ conducted a clinical trial that EES immediately enhanced forward reaching in two patients with similar SCI levels. One participant received stimulation at 2.0–6.5 V and 210 µs pulse width, while another participant used 2.9–3.0 V and 200–400 µs. At present, there are no better ways than doing a lot of pre‐tests to find out the effective parameters for each patient.

**Figure 1:**
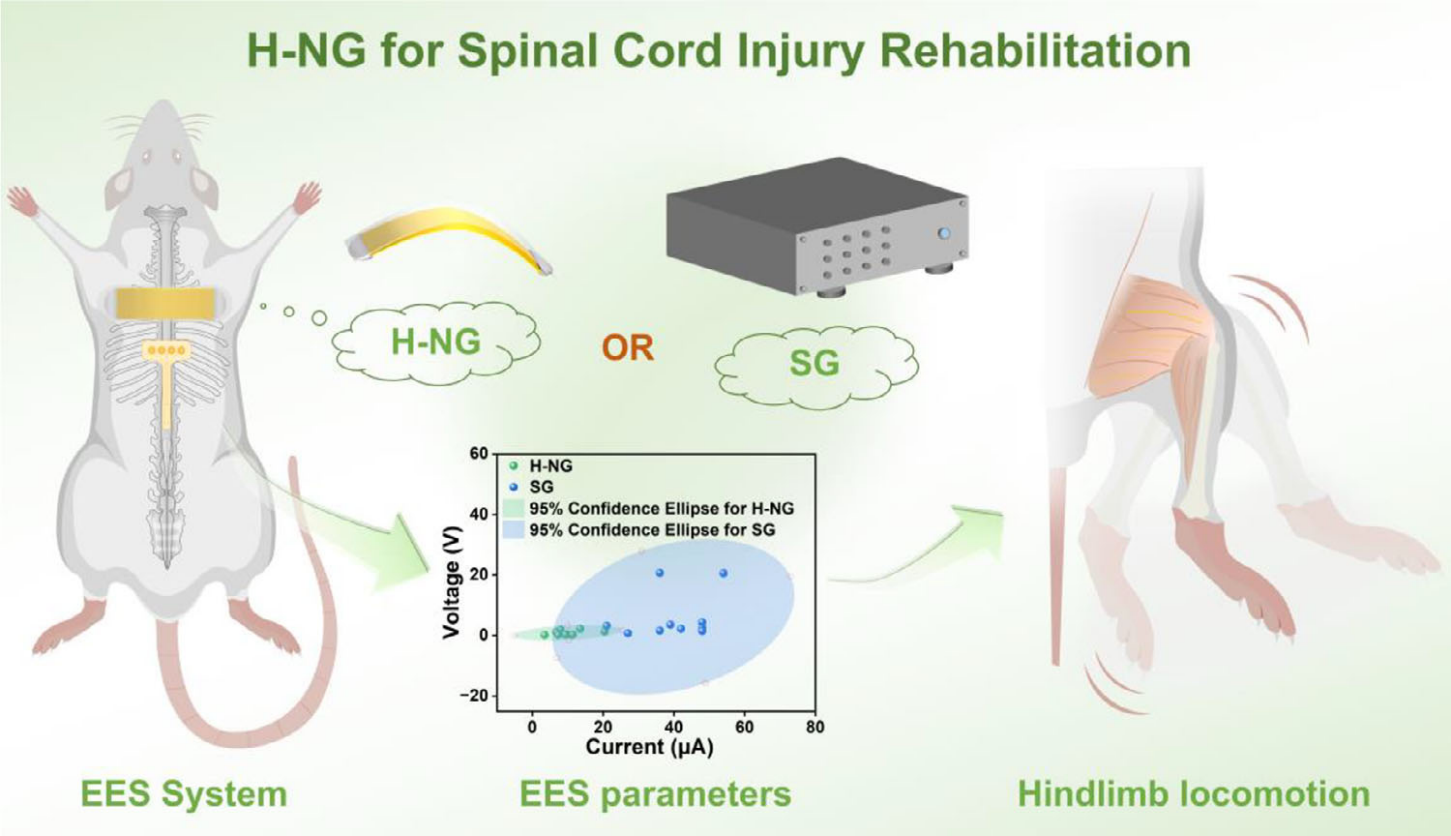
Schematic illustration of the implantable EES system based on the H‐NG.

In this study, we have developed a hybrid nanogenerator (H‐NG) consisting of both triboelectric and piezoelectric parts and investigated its efficacy in EES (as shown in **Figure** **1**). Electrodes are positioned outside the dura mater of the spinal cord in rats, and the H‐NG is implanted subcutaneously on the back. The H‐NG can be activated by finger squeeze to generate electrical currents and voltages in vivo. The H‐NG‐generated currents and voltages are capable of eliciting hindlimb locomotion in the rats. The effective H‐NG EES parameters and the electromyogram (EMG) signals of the hindlimb are all recorded. The results reveal that, in contrast to a traditional constant‐current stimulus generator (SG), the H‐NG could initiate hindlimb locomotion with significantly lower current and voltage. Moreover, the H‐NG exhibits much smaller individual differences in effective parameters to induce hindlimb locomotion in different rats. These advancements of the H‐NG are anticipated to facilitate the development of self‐powered, personalized, and more efficient EES therapies for SCI.

## 2 Structural Design and Characteristics of the H‐NG

**Figure 2:**
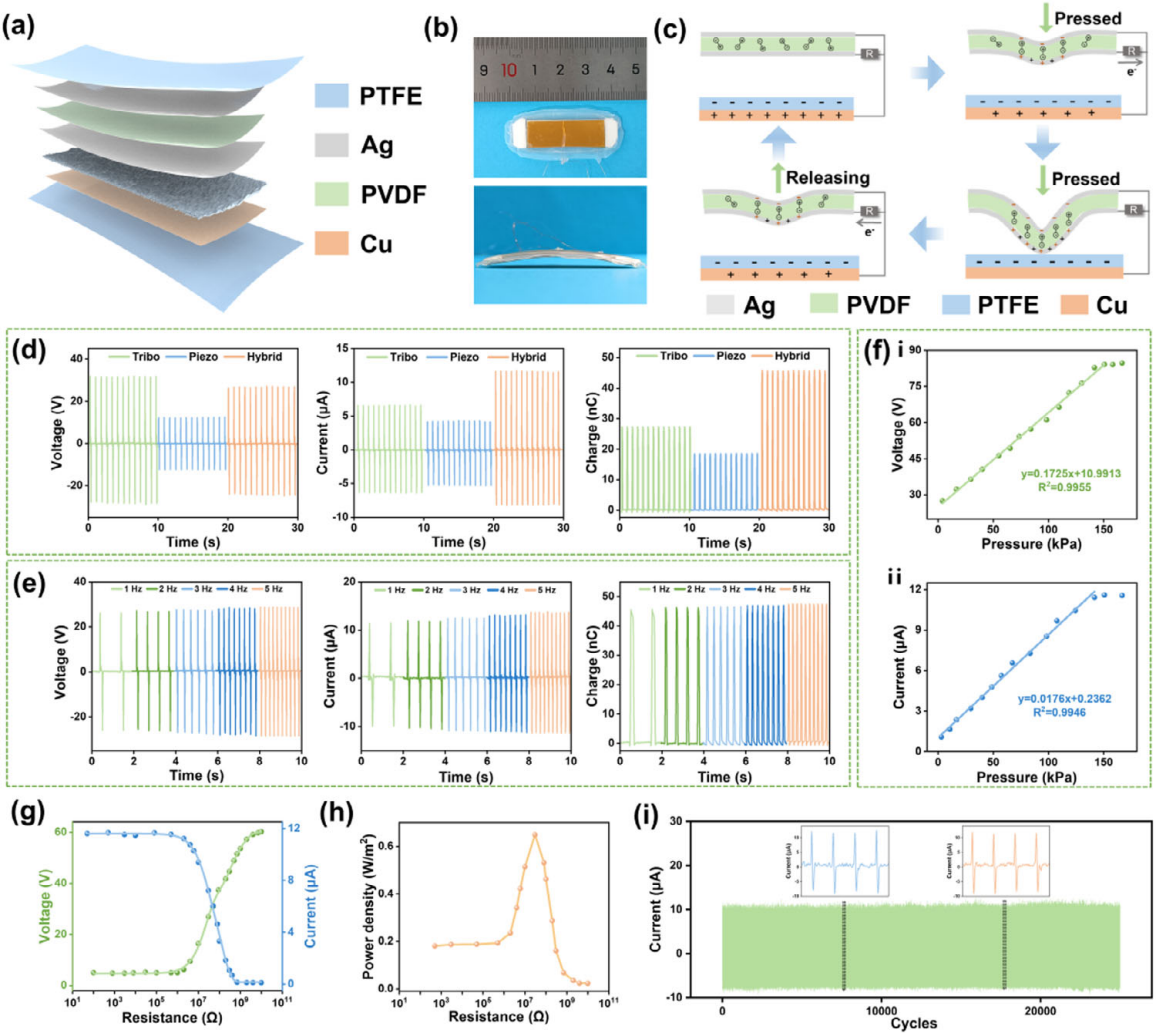
Structural design and electrical performance of the H‐NG. a) Schematic illustration of the H‐NG. b) Optical images of the H‐NG. c) Schematic diagrams of the working principle of the H‐NG. d) Outputs of the H‐NG with different parts, including *V_oc_*, *I_sc_*, and *Q_sc_*. e) Frequency‐response characteristics of the H‐NG under loading frequencies from 1 to 5 Hz. f) Linear fitting of peak‐to‐peak voltage, current, and pressure change. The variation of g) peak voltage, peak current, and h) peak power density of the H‐NG with different external load resistance. i) The stability test of the H‐NG under continuous operation for 25 000 cycles.

The H‐NG device is comprised of piezoelectric, triboelectric, and encapsulation components (**Figure** **2**a). Polarized PVDF with a high piezoelectric coefficient and coated with Ag thin film on both sides serves as the core piezoelectric part. Then the lower side Ag surface and a PTFE film are selected as the tribo‐positive and tribo‐negative layers respectively due to their notable triboelectric polarity difference, and a Cu film is attached to the other side of PTFE to transfer the induced charges. By combining piezoelectric and triboelectric effects, this device could convert mechanical energy into electrical form with a higher efficiency. To enhance the output performance of the H‐NG even further, the tribo‐surface of PTFE is polished with 3000# grit sandpaper in the same direction several times to yield a nanostructured surface (Figure S1, Supporting Information). This process increases the effective contact area between triboelectric layers, thereby boosting charge transfer. The relationship between the number of polishing times and device output is shown in Figure S2 (Supporting Information), which shows polishing five times generates the highest output. This is most likely because if polishing is more than five times, the microstructure will be destroyed and the actual contact area reduced. To shield the device from external environmental interference, PTFE films are utilized as encapsulation layers. The functional layers of the device are dimensioned at 3 cm × 1 cm, while the overall device is 4.5 cm × 1.5 cm (Figure 2b). This implantable device is fabricated with an arched configuration, which could conform more closely to the shape of the rat's back.

The working principle of the H‐NG device is illustrated in Figure 2c, as a combination of triboelectric and piezoelectric effects. In the initial state, as the top layer is away from the bottom layer, there is no triboelectric or piezoelectric potential among the various electrodes. When the force is applied to the H‐NG, the PVDF film bends and results in a piezoelectric potential on its surfaces. Meanwhile, the curved Ag and PTFE layers contact to cause charge transfer due to the triboelectric effect. As the applied force intensifies, both the contact between the triboelectric layers and the stress on the PVDF increase. Upon full contact between the top and bottom layers, the system achieves a new equilibrium in that both the triboelectric and piezoelectric potentials reach their peak levels. Therefore, the current will be induced during the deformation of the H‐NG if these different electrodes are connected through an external circuit. When the force is released, the curved PVDF and PTEF films begin to recover their original states, causing the electrons to be driven back to the upper layer.

To investigate the electrical output performance of the H‐NG in vitro, a linear motor is employed to provide mechanical stimuli. The H‐NG exhibits an open‐circuit voltage (*V_oc_*) of 26.5 V, short‐circuit current (*I_sc_*) of 11.6 µA, and transferred charge (*Q_sc_*) of 45.7 nC (Figure 2d). Additionally, the outputs of the single piezoelectric or triboelectric mode are also displayed. The device's performance is investigated in a frequency range from 1 to 5 Hz (Figure 2e), which indicates that the *V_oc_*, *I_sc_*, and *Q_sc_* outputs remain almost unchanged as the frequency increases. The mechanical response of the device is a critical attribute considered. As depicted in Figure 2f, the H‐NG exhibits outstanding linearity in voltage, current, and applied force, thereby confirming its high maneuverability. Further evaluation of the effective electrical power of the H‐NG shows that the peak voltage increases while the current decreases with increasing external resistance (Figure 2g), with the maximum peak power density obtained at ≈30 MΩ (Figure 2h). Meanwhile, the H‐NG demonstrates remarkable stability and resilience, maintaining an output current at 100% of its initial level after 25 000 working cycles (Figure 2i).

## 3 EES Enabled by the H‐NG to Control Hindlimb Locomotion

EES, an electrical neuromodulation method, has demonstrated critical roles in facilitating functional restoration after SCI. Through strategic placement of epidural electrodes at the proper site, EES can effectively enhance motor plasticity by doing rehabilitation. In this part, the ability of the H‐NG to elicit EES activation of hindlimb locomotion is investigated.

**Figure 3:**
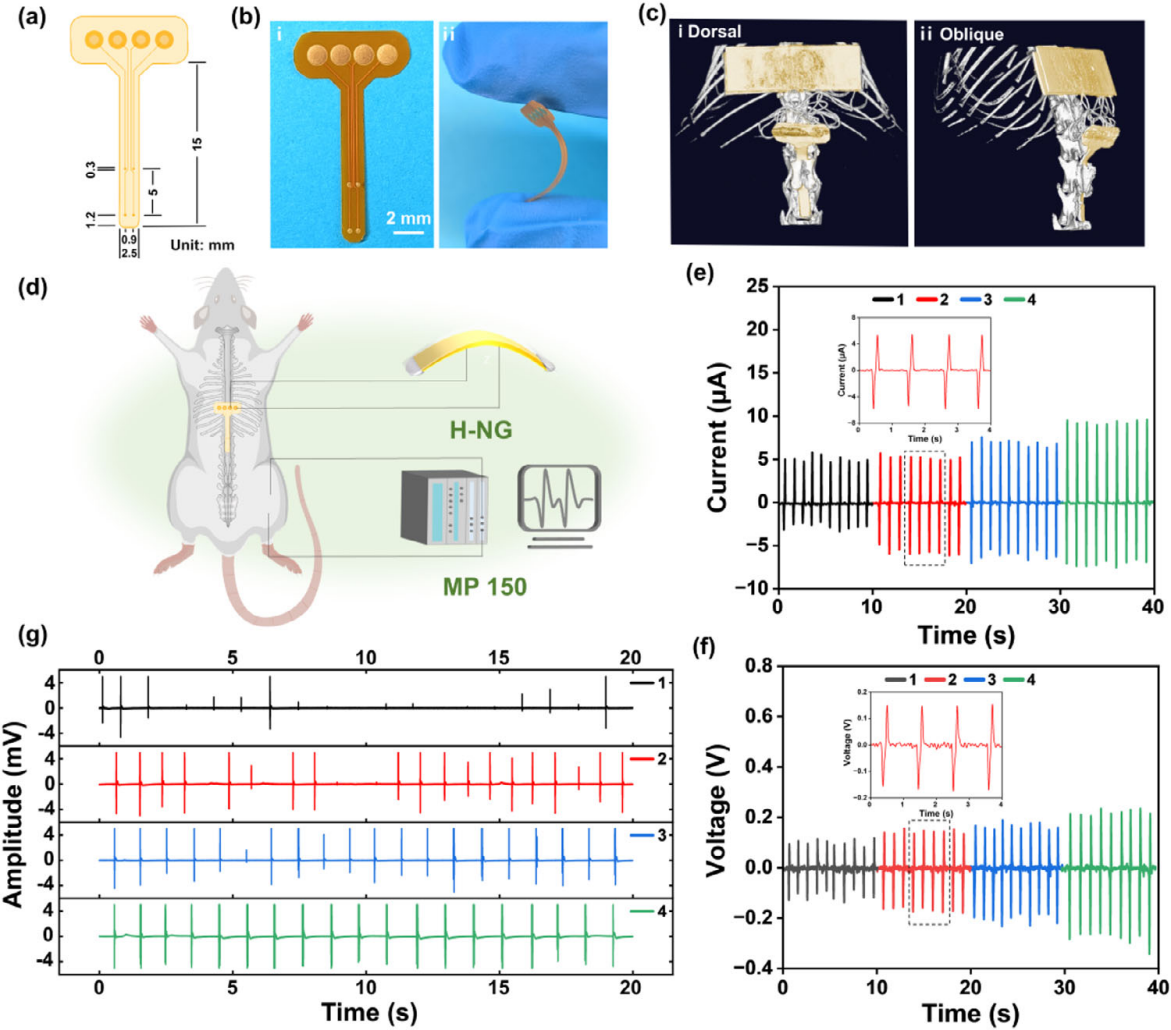
Efficacy of the H‐NG‐driven EES to induce hindlimb locomotion. a) Schematic diagram of the four‐electrode paddle. b) Optical image of the four‐electrode paddle in (i) normal state and (ii) bent state. c) CT images of the implantable EES system based on the H‐NG and the four‐electrode paddle from (i) a dorsal view and (ii) an oblique view of the rat. d) Schematic diagram of the EES system based on the H‐NG and the hindlimb EMG signals measurement system. e) Graded currents measured in the circuit. f) Graded voltages measured across the stimulation electrodes on the rat's spinal cord. g) EMG signals of the rat's hindlimb.

A flexible four‐electrode paddle serves as the stimulation electrode for EES, as illustrated in **Figure** **3**a. This electrode is fabricated on a polyimide (PI) substrate and there are four exposed Au contact electrodes to do the stimulation. The conductive metal wires are encapsulated by another layer of PI. Upon connecting to the power device, the respective region between the Au electrodes on the spinal cord will be subjected to electrical stimulation. This process can induce locomotion in the corresponding left or right hindlimb. The electrode paddle, measuring 1.5 cm × 3 cm (Figure 3bi), with a thickness of 60 µm, exhibits high flexibility (Figure 3bii), benefiting its contact with the spinal cord. The rats’ lumbosacral spinal cord (T13‐L1 segment) is exposed, and the tail of the paddle is inserted between the spinal vertebra and the dura mater, ensuring direct contact of the exposed Au electrodes with the spinal cord.

An EES system is constructed based on the electrode paddle and our H‐NG device (Figure S3, Supporting Information). The H‐NG is implanted subcutaneously on the rat's back, and its arched configuration accommodates the rat's back anatomy precisely. The H‐NG device is connected to the conductive metal terminal on the EES paddle. The configuration of the implanted system is visualized using computerized tomography (CT), with the implant highlighted as a golden region (Figure 3c). Through applying external manual force (repeated finger squeezing) to the implanted H‐NG device, the induced electrical signals are conducted to the spinal cord, and the electrical signals are able to elicit the locomotion of the rat's hindlimb (Video S1, Supporting Information). Employing the H‐NG to drive ESS of the spinal cord brings in self‐powering ability, control convenience, and power sustainability.

To investigate the H‐NG‐enabled EES more precisely, the H‐NG is driven outside the rat's body to elicit graded hindlimb locomotion and EMG signals (Figure 3d; Video S2, Supporting Information). The voltage applied to the rat's spinal cord is precisely measured using an oscilloscope, and the current within the circuit is accurately monitored using an electrometer. EMG signals are recorded by a physiological signal recorder. As shown in Figure 3e,f, with an increase in the manual force on the H‐NG, the peak current in the circuit increases from 5.55 to 9.30 µA and the peak voltage applied on the rat from 0.13 to 0.29 V. Regarding the EMG signals, there are not significant differences in the EMG amplitude among the four groups, but there are obvious differences in the emergence frequency in the EMG signals (Figure 3g). Under a low current, the EMG signals feature randomly appeared peaks, but some peak intensity can be quite high (up to 5 mV, the saturated level). With the increase in the current, the incidence of high EMG peaks increases, culminating in a stable and consistent emergence of EMG signals.

## 4 EES Enabled by Traditional SG to Control Hindlimb Locomotion

**Figure 4:**
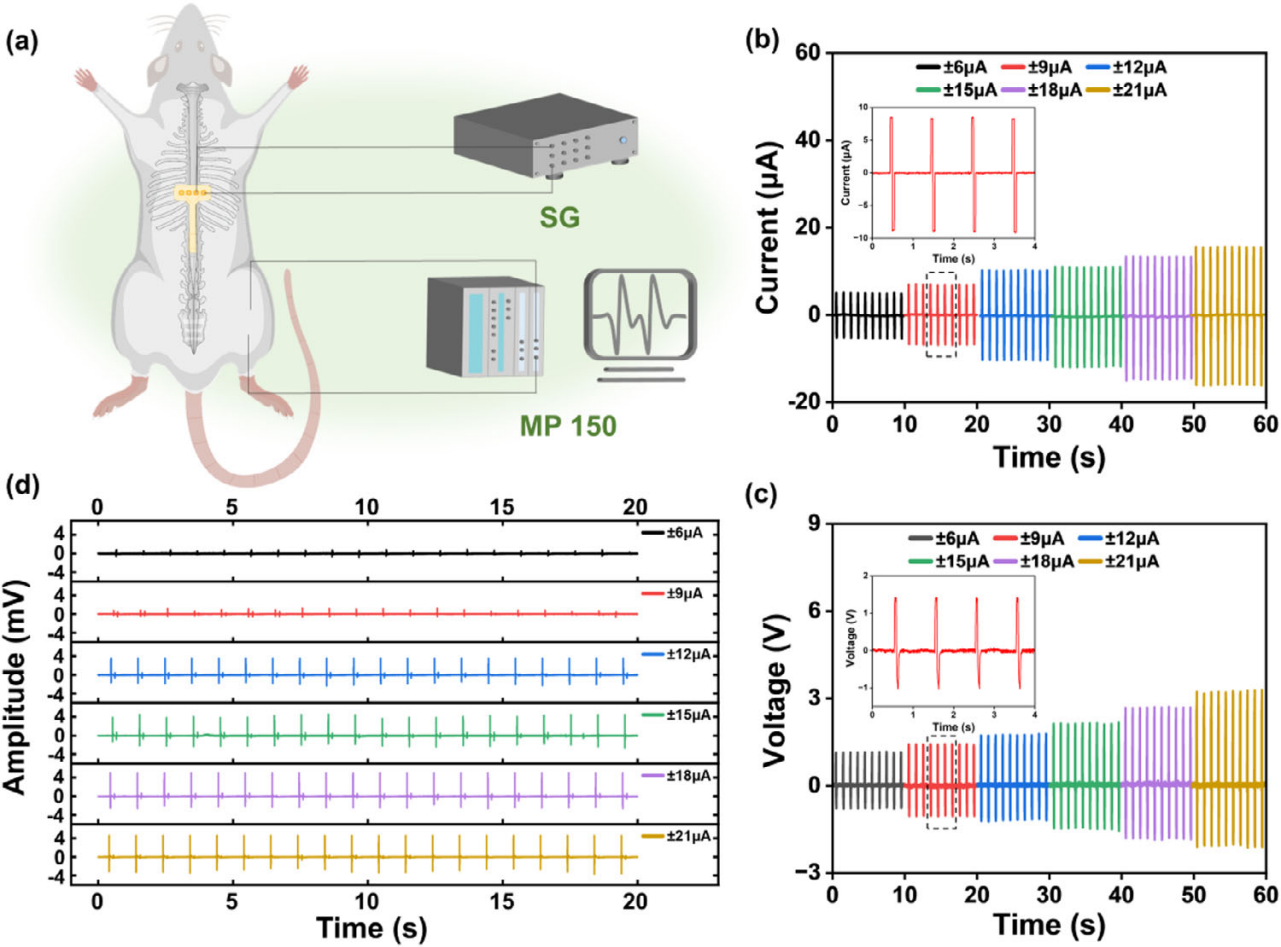
Efficacy of the SG‐driven EES to induce hindlimb locomotion. a) Schematic diagram of the EES system based on the SG and the hindlimb EMG signals measurement system. b) Graded currents are measured in the circuit. c) Graded voltages were measured across the stimulation electrodes on the rat's spinal cord. d) EMG signals of the rat's hindlimb.

Next, we want to find out the differences between the H‐NG‐driven EES and the traditional SG‐driven EES. However, most previous investigations on EES parameters are high‐frequency stimulations, extending from 40 to 300 Hz.^[^ ^23^, ^26^, ^27^, ^28^, ^29^, ^30^ ^]^ Therefore, a constant‐current SG is employed in our study at the same low frequency as the H‐NG for a fair comparison. The constant‐current SG is set to generate square‐wave alternating current at 1 Hz with a pulse width of 100 ms (Figure S4, Supporting Information). The pulse width is chosen to be 100 ms because, in this setup, the total charge in one cycle would be appropriately the same for the SG and the H‐NG, which enables an easy comparison based on the peak current intensity. The circuit current, the corresponding voltage applied on the rat, and the EMG signals of the hindlimb are quantitatively measured in the same way above (**Figure** **4**a; Video S3, Supporting Information). As shown in Figure 4b,c, upon escalation of the applied current from 6 to 21 µA, the voltage applied on the rat's spinal cord rises from 1.1 to 3.2 V. At the current of 6 µA, the rat's hindlimb initiated a response pattern of low intensity and full frequency (Figure 4d). As the current increases, the EMG signals become apparent at 9 µA, and there is a progressive enhancement in the EMG signal intensity correlating with the increase of the current. A saturated EMG is reached at 21 µA with an amplitude of 5 mV. Notably, some EMG signals exhibited bimodal peak patterns, with a time interval of ≈150 ms between the two peaks. It is speculated that the cause is the multiple conduction pathways of neural signals and the dual innervation attributes of some muscle groups.^[^ ^31^, ^32^ ^]^

## 5 Statistical Analysis and Individual Differences Between H‐NG and SG

**Figure 5:**
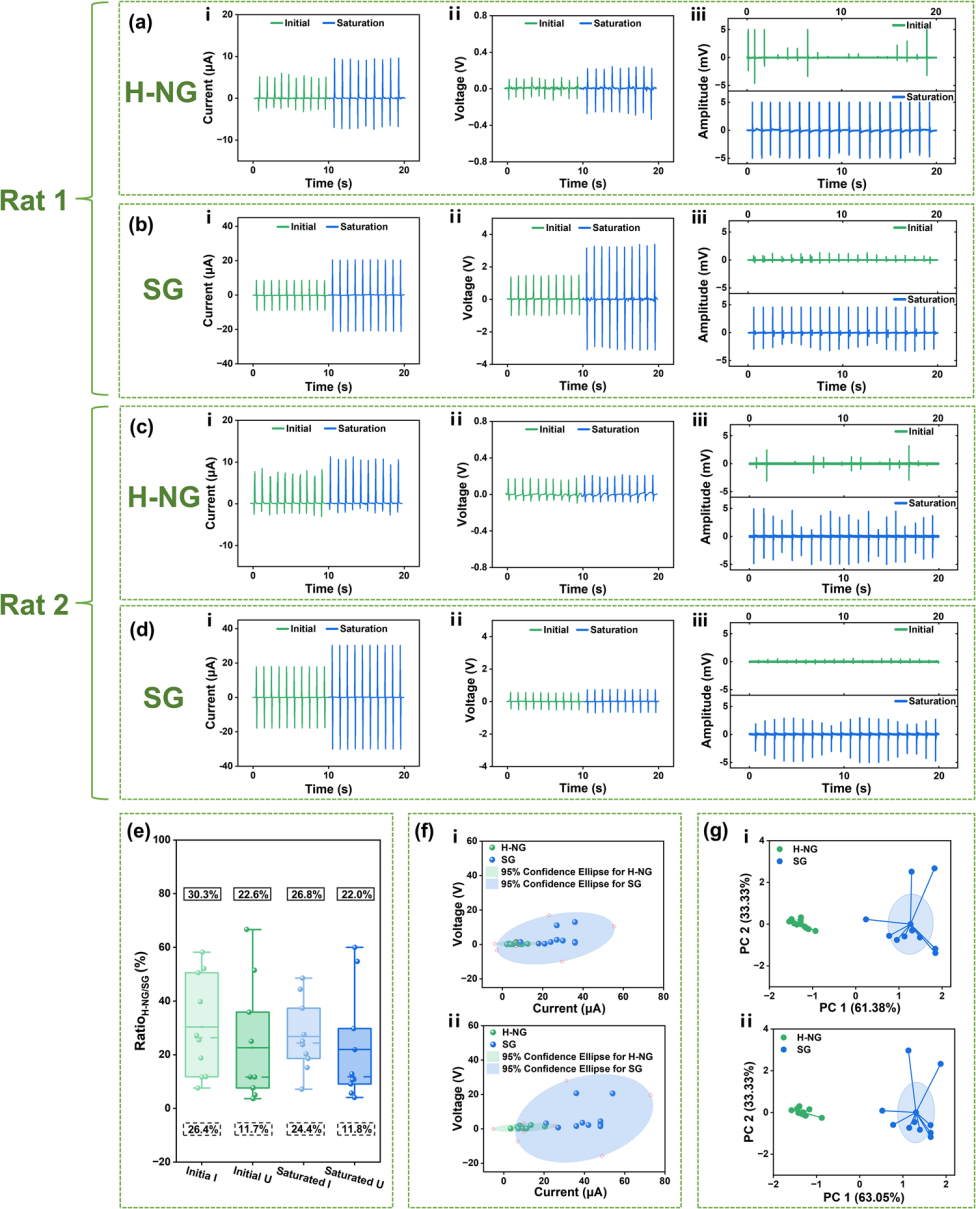
Comparison and analysis of EES parameters of the H‐NG and the SG. The current, voltage, and EMG signals of the hindlimb under the H‐NG a, c) and SG b, d) stimulation from Rat 1, and Rat 2, respectively. e) Box plot of the ratio of EES parameters between the H‐NG and the SG. The mean is in the solid line box and the median is in the dashed line box. f) Data scatter plots under the H‐NG and the SG stimulation, including (i) initial values ​​and (ii) saturated values. g) Data cluster analysis under the H‐NG and the SG stimulation, including (i) initial values ​​and (ii) saturated values.

In all, 10 rats are studied for both the H‐NG‐driven EES and SG‐driven EES to conduct a comparative analysis of the two power devices. For simplification, only the initial and saturated EMG signals are extracted to do statistical analysis. **Figure** **5**a,b is the extracted data of the current, voltage, and EMG signals from the rat in Figure 4 (Rat 1). Figure 5c,d are the data from Rat 2. More data can be found in Figure S5 (Supporting Information). The data from all 10 rats are analyzed in Figure 5e–h.

Figure 5a–d illustrate that the H‐NG exhibits markedly lower voltage and current than the SG, to induce both the initial and saturated EMG signals. Additionally, the initial EMG signals of the H‐NG are noted for absent or very weak peaks, occasionally interspersed with peaks of high intensity. In contrast, the initial EMG signals of the SG feature consistently low peak intensity and regular peak frequency. The saturated EMG signals of the H‐NG and SG show a similar pattern with a stable occurrence frequency and high signal intensity. Furthermore, different rats exhibit diverse characteristics of EES. For example, the current and voltage of Rat 2 demonstrate asymmetry between the positive and negative peaks, although the integral area of the peaks is equivalent for both polarities. Besides, the peak amplitude of the saturated EMG signal in Rat 2 exhibits a periodic pattern, in both the H‐NG and the SG stimulations. This periodic peak amplitude of EMG signals, to the best of our knowledge, has not been previously reported. This is primarily because the majority of previous studies have employed EES with a high frequency (for example, 300 Hz), not the low frequency of 1 Hz utilized in our study, thereby lacking comparable reference data and analyses.

Figure 5e depicts the H‐NG/SG ratio of the EES parameters. The quantitative results show that to elicit initial EMG, the average ratio of the H‐NG current over the SG current is 30.3%, and the average voltage ratio is 22.6%. To elicit saturated EMG, the average current ratio of H‐NG/SG is 26.8%, and the average voltage ratio is 22.0%. These findings indicate that to induce the same extent of locomotion, the required current and voltage for the H‐NG are only ≈1/4 to 1/3 of that of the SG. The H‐NG can elicit a comparable EES effect to that of the SG with significantly reduced power requirement, which not only conserves energy consumption but also diminishes potential harm to tissues and organs. However, the variations of the ratios are large. For the initial and saturated EMG, the median current ratios of H‐NG/SG are 26.4% and 24.4%, respectively, lower than the average ratios. In addition, the median voltage ratios of H‐NG/SG to elicit the initial and saturated EMG ​​are only 11.7% and 11.8%, respectively, remarkably beneath the average values. These results suggest that the individual differences of the EES are unignorable.

To look into the individual differences even further, scatter and cluster comparative diagrams focusing on the EES parameters are plotted. Figure 5f (i and ii) demonstrate the distribution state of voltages and currents corresponding to the initial and saturated EMG signals, respectively. The current‐voltage points from 10 rats are represented in green (H‐NG) and blue (SG), respectively, with 95% confidence ellipse plotted. The lengths of the major and minor axes of the ellipse indicate the data's dispersion, with the major axis length corresponding to the direction of maximum variability of the data. Accordingly, the EES current shows more remarkable variability than the voltage. Specifically, the ellipse center represents the mean of the two variables, current and voltage. For the initial EMG, the central parameter pairs are (6.83 µA, 0.17 V) for the H‐NG, and (23.61 µA, 3.63 V) for the SG, respectively. The corresponding central parameter pairs are (10.11 µA, 0.82 V, H‐NG) and (26.97 µA, 4.76 V, SG) for the saturated EMG. The results suggest again that the EES parameters required for the H‐NG are much lower than the SG. Besides, the area of the confidence ellipse signifies the potential range of the joint distribution of current and voltage at a 95% confidence level. Notably, the area of the green ellipse is remarkably smaller than that of the blue, indicating a much narrower distribution range for the EES parameters of H‐NG, which in turn suggests a significant reduction in individual differences.

Subsequently, we employed principal component analysis (PCA) to preprocess the EES parameters of 10 rats, aiming at dimensionality reduction to enhance both clustering efficacy and analytical precision. Following this, the K‐means algorithm is applied to facilitate the supervised classification of the current–voltage points for the H‐NG and the SG. The current parameter emerged as the first principal component (PC 1) with a contribution rate exceeding 60% both in the initial and saturated EMG signals, while voltage is the second principal component (PC 2) at 33.33%. This suggests that the current parameter more effectively captures the variability within the dataset. The inter‐cluster distance indicates the dissimilarity between the two groups. Therefore, the notable separation between the H‐NG and the SG clusters signifies a substantial distinction in their feature space representation. The centroids of these clusters represent the central tendency of each group, encapsulating the typical values across variable dimensions. The proximity of data points to their respective centroids indicates the internal homogeneity of each cluster. The results suggest that current–voltage points for the H‐NG are significantly closer to their centroid, suggesting a higher degree of clustering and similarity among data points. In contrast, data points for the SG exhibit more pronounced variability, indicating a lower level of clustering cohesion. These outcomes prove that as a power device, the H‐NG effectively mitigates individual differences for EES, which shows the prospect of facilitating the advancement of EES in the treatment of SCI.

## 6 Discussion and Conclusion

Although EES has been demonstrated effective in human applications, its complexity inherently and high energy requirements add an extra burden on SCI patients. Therefore, the improvements of EES devices and efficiencies are highly demanded to improve treatment outcomes. The H‐NG, bringing in self‐powering ability, control convenience, and power conservation for EES, is anticipated to advance self‐powered, personalized, and more efficient therapies for SCI patients.

The EES frequency used in the previous studies mainly concentrated in a range of 40 to 300 Hz. However, these do not align with the natural rhythm of human or animal locomotion. In this study, the efficacy of a low frequency of 1 Hz is investigated. Comparative analysis reveals that the H‐NG can exert the spinal cord and elicit hindlimb locomotion in rats, with a similar effect to the SG but much‐reduced parameters. This indicates that the H‐NG can contribute to the energy efficiency. Furthermore, statistical analysis illustrates that the EES parameters of the H‐NG are more concentrated, suggesting that as a power source for EES, the H‐NG effectively minimizes individual differences. Meanwhile, the mechanically driven characteristics of H‐NG allow for self‐regulated stimulation intensity and potential personalized EES therapy.

Overall, the H‐NG has demonstrated promise in EES as a therapeutic strategy for SCI. The benefits of the H‐NG's self‐driven operation, high energy efficiency, and much reduced individual differences, robustly support the viability of integrating NG into the clinical EES strategy. Nevertheless, certain aspects deserve to be investigated further, including the underlying mechanism of the phenomenon we have discovered, the optimization of the electrode‐spinal cord interface, and the accuracy of hindlimb locomotion control. Moreover, the physiological disparities between humans and rats are significant, and the long‐term effect of the H‐NG applied in EES requires assessment. Furthermore, the human body's internal environment is more intricate, thus bringing more challenges to the device's output, stability, and efficacy of EES. In brief, a comprehensive grasp of the fundamental mechanism, coupled with extensive and prolonged research, is imperative for the clinical implementation of the H‐NG in conjunction with EES as a promising SCI treatment technology.

## 7 Experimental Section

### Fabrication of the H‐NG

The prepared H‐NG consists of the piezoelectric and triboelectric parts. A commercial polarized PVDF film (52 µm) was used as the piezoelectric material, with Ag electrodes coated on both sides. The PVDF film was cut into 3 cm × 1.5 cm and Cu wires were connected on both sides. The PTFE film was cut into the same size and polished several times in the same direction with 3000#‐grit sandpaper as the tribonegative layer. The Cu tape was attached to the other side as the electrode and a Cu wire was connected. The two parts above were connected with a spacer. PET substrates were attached to the top and bottom as support, and the device was finally encapsulated with PTFE tapes.

### Characterization of the H‐NG

To obtain the triboelectrical properties of the device, a linear motor (LinMot E1100) was employed to provide periodic contact separation motion. The open circuit voltage (*V_oc_*), short circuit current (*I_sc_*), and transfer charge (*Q_sc_*) of the H‐NG were recorded through an electrometer (Keithley 6517B) and recorded by an oscilloscope (Teledyne LeCroy HD 4096).

### Animal Experiments

All animal experiments were conducted under ethical approval from the Committee on Ethics of the Beijing Institute of Nanoenergy and Nanosystems (2024027LZ). Ten‐week‐old female Sprague‐Dawley rats were obtained from the Beijing Vital River Laboratory Animal Technology Co., Ltd. The anatomical location of the T13 and L1 spinal cord was first exposed, and then some spinal backbone between the two segments was removed to expose a 1 mm gap. The tail part of the 4‐electrode paddle was inserted through the gap and the stimulation electrodes were placed between L1 and L2 segments. The conductive points at the top were connected to the Stimulus Generator (AD Instruments, Multi‐Channel, STG4008) or the H‐NG through Cu wires to provide ES. EMG signals were recorded by MP150 (Biopac, USA). The positive record electrode was inserted into the hindlimb muscle of the rat, the negative record electrode was inserted subcutaneously at the ankle, and the ground electrode was placed subcutaneously away from the measurement site. For the implantable EES system consisting of the H‐NG and the electrode paddle, the H‐NG was implanted subcutaneously on the rat's back. The image of the implantable EES system was obtained by micro‐CT.

### Data Analysis

Upon ESS of the rat's spinal cord, the first detection of a hindlimb locomotion signal was identified as the initial EMG signal. As the hindlimb locomotion intensifies and reaches its maximum without further increase, the first occurrence of maximum intensity was designated as the saturated EMG signal.

All statistical data were analyzed using Origin software. In the box plot, the term Ratio_H‐NG/SG_ denotes the comparative ratio of EES parameters associated with the EMG signals elicited by H‐NG and SG stimulation. Specifically, the current ratio for the initial EMG signal was termed Initial I, while the voltage ratio was Initial U. Similarly, the current ratio for the saturated EMG signal was defined as Saturated I and the corresponding voltage ratio was Saturated U. In the scatter plots, the 95% confidence ellipse refers to the elliptical area that contains 95% of the probability of data point distribution. Its area was positively correlated with the degree of dispersion of the data. In the cluster plots, the EES parameter data points have been subjected to preprocessing via PCA, followed by supervised clustering utilizing the K‐means algorithm. The current parameter was designated as PC 1, while the voltage parameter constituted PC 2. The current‐voltage parameters associated with the initial and saturated EMG signals were then plotted individually.

PCA involves the calculation of the covariance matrix, the formula is:

(1) $$Cov\left(X\right)=1/\left(m-1\right)\cdot \left(X^{T}X\right)$$

where *X* is the standardized data matrix and *m* is the number of samples.

Then the eigenvalues ​​and eigenvectors of the covariance matrix were calculated. The eigenvector indicates the direction of the principal component within the dataset, constituting a new coordinate axis. The eigenvalue represents the variance of the data along the direction of the corresponding eigenvector. A larger eigenvalue signifies a stronger ability to explain the principal component's data. Project the original data onto the selected principal component to obtain a reduced‐dimensional data set. This was accomplished by multiplying the original data matrix *X* with the selected principal component matrix as follows:

(2) $$X_{new}=X\cdot V_{k}$$

where *V_k_* is the matrix containing the first *k* vectors. Following the PCA processing, the data were further analyzed using the K‐means clustering algorithm. For the EES parameters for both the initial and saturated EMG signals, the dataset was partitioned into two distinct clusters based on the power device (H‐NG or SG). The objective of the K‐means algorithm was to minimize the sum of the squared Euclidean distances from each data point to the centroid of its respective cluster, as delineated by the following equation:

(3) $$d=\sqrt{{\left(x_{2}-x_{1}\right)}^{2}+{\left(y_{2}-y_{1}\right)}^{2}}$$

where (*x_1_*, *y_1_*) and (*x_2_*, *y_2_*) are the coordinates of the two data points.

The sum of the squared errors (SSE) is used to assess the quality of clustering. The goal of the algorithm is to minimize this value.

(4) $$SSE=\sum_{k=1}^{K}\sum_{x_{i}\in C_{k}}d{\left(x_{i},C_{k}\right)}^{2}$$

where d(*x_i_*, *C_k_*) is the distance from the data point *x_i_* to the centroid *C_k_* of the cluster.

## Conflict of Interest

The authors declare no conflict of interest.

## Supporting information



Supporting Information

Supporting Information

Supporting Information

Supplemental Video 1

Supplemental Video 2

Supplemental Video 3

## Data Availability

The data that support the findings of this study are available in the supplementary material of this article.
