# Supplementary material for: Nanogenerator Neuromodulation to Enable Locomotion Rehabilitation for Spinal Cord Injury via Epidural Electrical Stimulation
Source: Adv Sci (Weinh). 2025 May 23;12(24):2501425. doi: 10.1002/advs.202501425 (PMC12199601; doi:10.1002/advs.202501425)
Supplement: Supplementary file 1 — Supporting Information [file ADVS-12-2501425-s003.docx]

Supporting Information

**Nanogenerator neuromodulation to enable locomotion rehabilitation for spinal cord injury via epidural electrical stimulation**

*Cong Li^#^, Yizhu Shan^#^, Shihao Zheng^#^, Puchuan Tan, Yuan Bai, Engui Wang, Lingling Xu, Ruizeng Luo, Shengyu Chao, Jing Huang, Pengyu Ren*, Zhou Li*, Hongqing Feng**


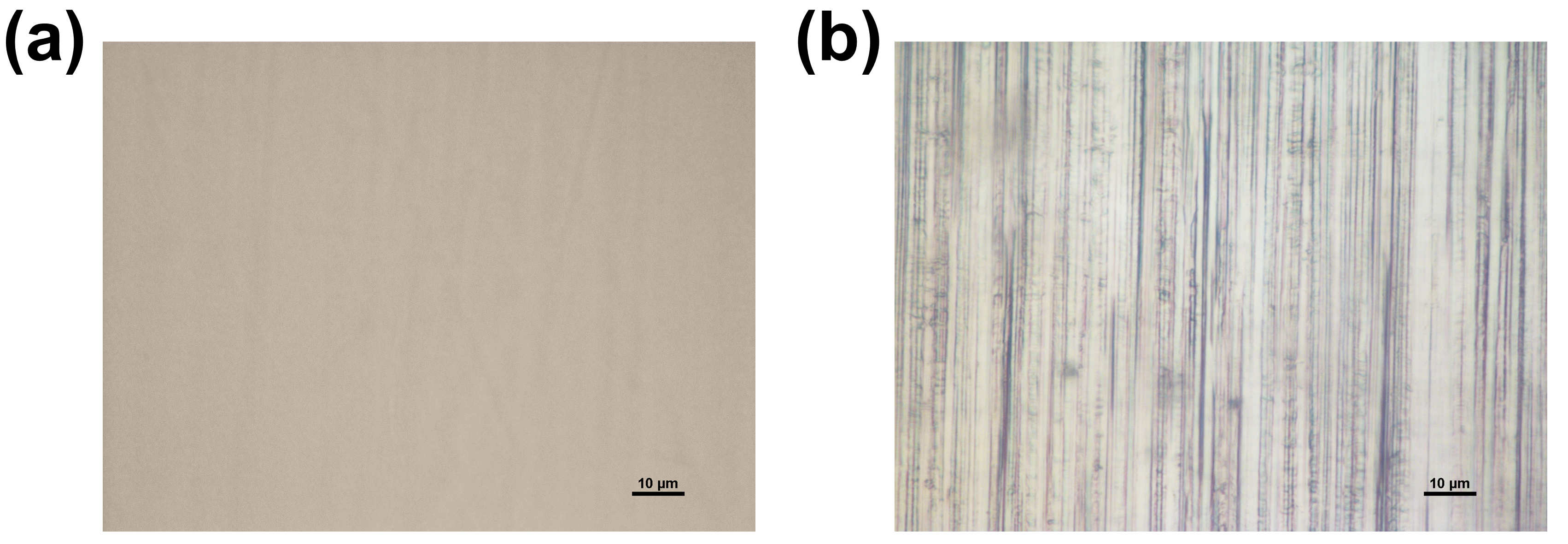


**Figure S1** Optical microscope images of (a) unpolished PTFE film and (b) PTFE film polished with sandpaper of grit size 3000# five times.


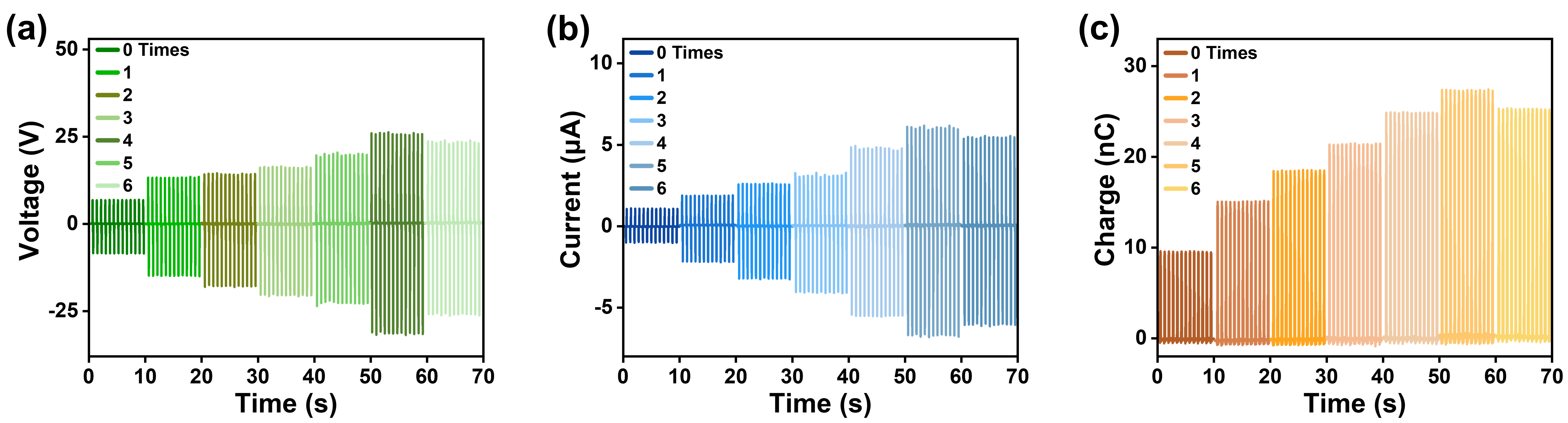


**Figure S2** The output (a) voltage, (b) current, and (c) transferred charges of the triboelectric part of the H-NG consisting of Ag and PTFE film polished zero to six times.


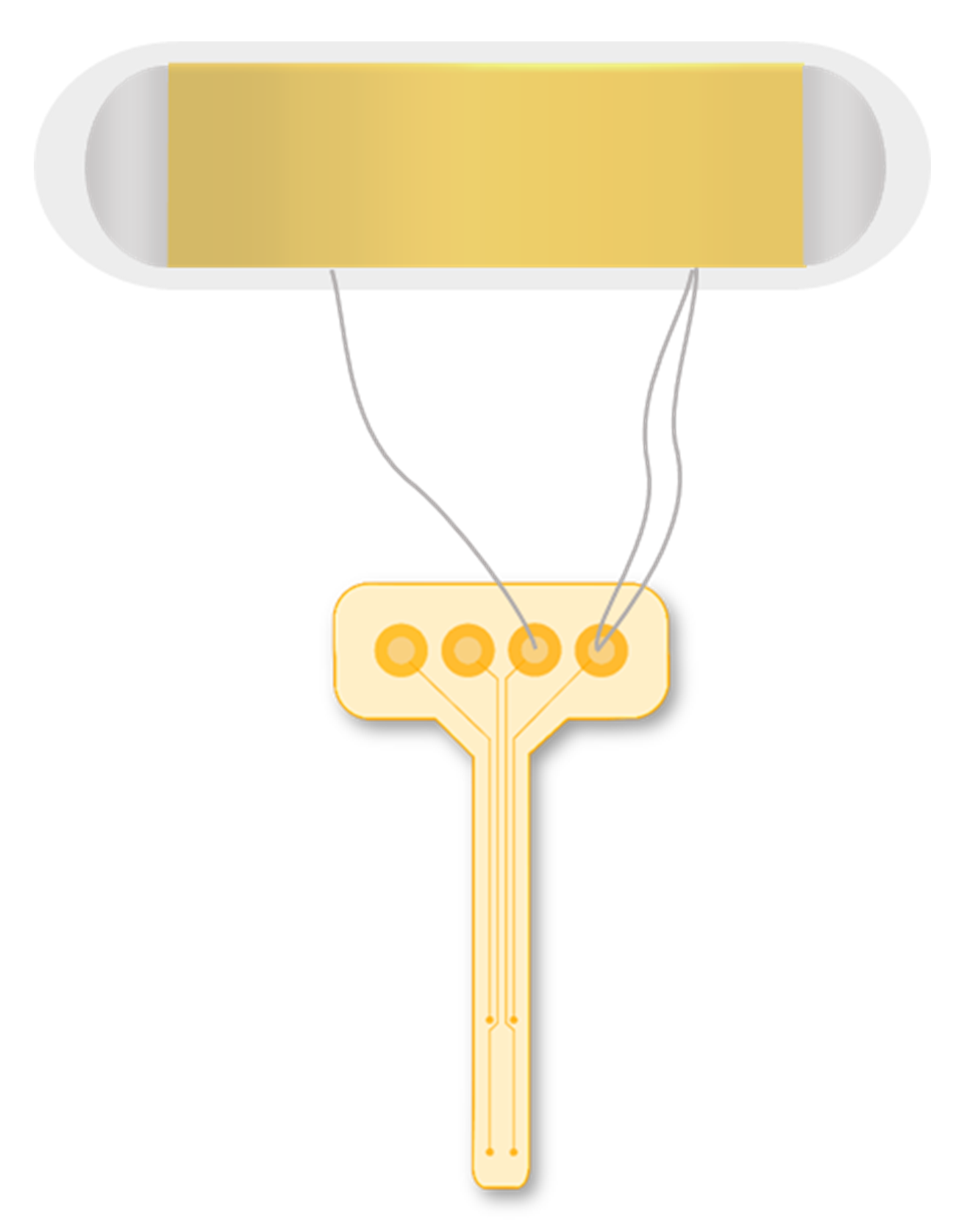


**Figure S3** Schematic diagram of the implantable EES system.


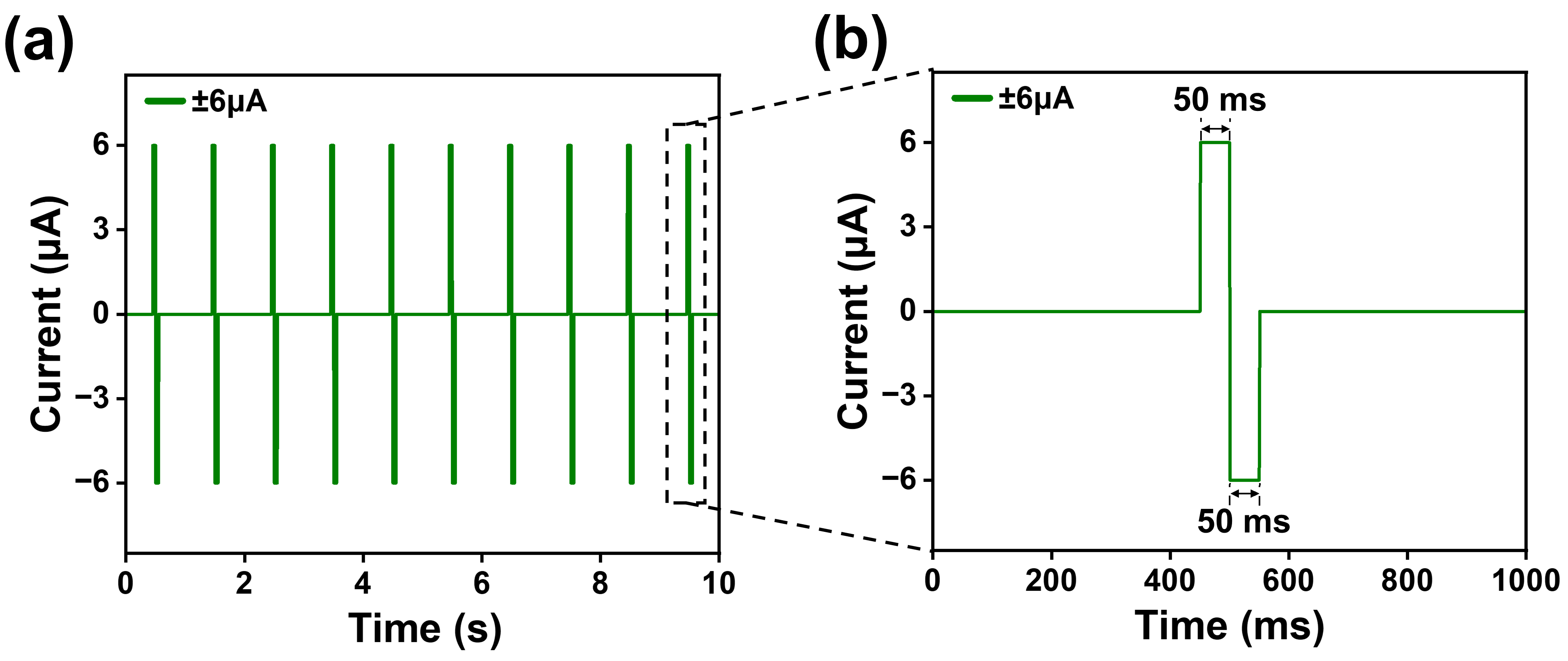


**Figure S4** The EES current parameters set on the SG (taking ±6 μA as an example). (a) A square wave with a frequency of 1 Hz. (b) An enlarged view of a current signal, with a pulse width of 100 ms.


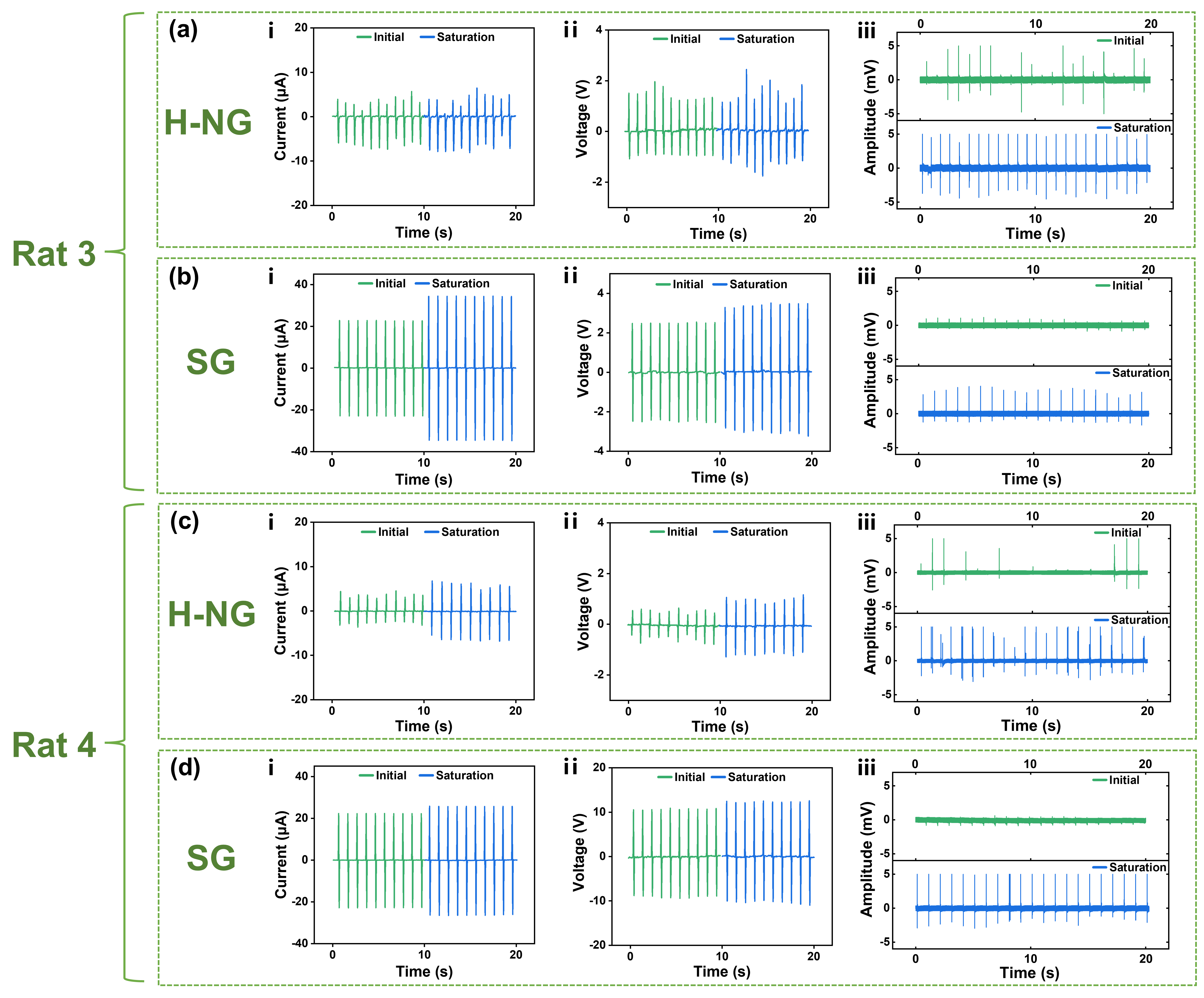


**Figure S5** Comparison of EES parameters of the H-NG and the SG. The current, voltage, and EMG signals of the hindlimb under the (a) H-NG and (b) SG-driven EES from Rat 3. The current, voltage, and EMG signals of the hindlimb under the (c) H-NG and (d) SG-driven EES from Rat 4.
